# A Randomized Clinical Trial on Comparing The Cycle Characteristics of Two Different Initiation Days of Letrozole Treatment in Clomiphene Citrate Resistant PCOS Patients in IUI Cycles

**DOI:** 10.22074/ijfs.2015.4204

**Published:** 2015-04-21

**Authors:** Nayereh Ghomian, Ashraf Khosravi, Nezhat Mousavifar

**Affiliations:** Ovulation Dysfunction Research Center, Faculty of Medicine, Mashhad University of Medical Sciences, Mashhad, Iran

**Keywords:** Letrozole, Clomiphene Citrate, Polycystic Ovarian Syndrome (PCOS)

## Abstract

**Background:**

There are still many questions about the ideal protocol for letrozole (LTZ) as the commonest aromatase inhibitor (AI) used in ovulation induction. The aim of this study is to compare the ultrasonographic and hormonal characteristics of two different initiation times of LTZ in clomiphene citrate (CC) failure patients and to study androgen dynamics during the cycle.

**Materials and Methods:**

This randomized clinical trial was done from March to November 2010 at the Mashhad IVF Center, a university based IVF center. Seventy infertile polycystic ovarian syndrome (PCOS) patients who were refractory to at least 3 CC treatment cycles were randomly divided into two groups. Group A (n=35) receiving 5 mg LTZ on cycle days 3-7 (CD3), and group B (n=35) receiving the same amount on cycle days 5-9 (CD5). Hormonal profile and ultrasonographic scanning were done on cycle day 3 and three days after completion of LTZ treatment (cycle day 10 or 12). Afterward, 5,000-10,000 IU human chorionic gonadotropin (hCG) was injected if at least one follicle ≥18 mm was seen in ultrasonographic scanning. Intrauterine insemination (IUI) has been done 36-40 hours later. The cycle characteristics, the ovulation and pregnancy rate were compared between two groups. The statistical analysis was done using Fisher’s exact test, t test, logistic regression, and Mann-Whitney U test.

**Results:**

There were no significant differences between two groups considering patient characteristics. The ovulation rate (48.6 vs. 32.4% in group A and B, respectively), the endometrial thickness, the number of mature follicles, and length of follicular phase were not significantly different between the two groups.

**Conclusion:**

LTZ is an effective treatment in CC failure PCOS patients. There are no significant differences regarding ovulation and pregnancy rates between two different protocols of LTZ starting on days 3 and 5 of menstrual cycle (Registration Number: IRCT201307096467N3).

## Introduction

Clomiphene citrate (CC) is known as one of the oldest drugs that has remained the standard choice for ovulation induction (1). CC has been an appropriate, non-expensive, and highly effective agent for inducing ovulation since 1963 (2). However, it certainly has not been successful in all patients; about 15-20% of women do not ovulate on CC, labeled as CC-resistant group (3). There are also other problems reported about CC, such as the anti-estrogenic mucosal and endometrial changes (2) that lead to higher rate of abortion and miscarriage in ovulatory women (3, 4).

Letrozole (LTZ), the prominent drug in the aromatase inhibitor (AI) family, has been introduced as a new choice for ovulation induction in the past decade, especially in polycystic ovarian syndrome (PCOS) patients who have failed to respond to CC. LTZ also seems to be very efficient in pregnancy rates, equivalent to injectable gonadotropins, at lower cost and with fewer adverse effects (5).

Furthermore, there are extra advantages for LTZ-therapy in comparison to CC, including: normal negative feedback mechanism for follicle- stimulating hormone (FSH) in the brain, more mono-follicular cycles, no negative antiestrogenic effects on the endometrial and cervical mucus, lower risk of ovarian hyperstimulation syndrome (OHSS), and lesser need for cycle monitoring (6).

By reviewing the literature, we found 2000 articles published related to CC since 1963, whereas, there is only about 200 articles published related to LTZ since 2000 (7).

Since LTZ is a new agent in the era of ovulation induction, there are several questions regarding the best protocol for administering. The usual doses for LTZ are mentioned as 2.5 and 5 mg. Doses higher than 5 mg per day for 5 days may result in persistence of aromatase inhibition that is followed by low estrogen level for normal endometrial development by the time of ovulation. Some researchers have suggested different LTZ protocols as follows: single dose of 20 mg given on cycle day 3, extended dose for up to 7-10 days, and step-up protocol including an escalating dose of 2.5 mg on day 3 along with 10 mg on day 6. The suggested starting day of LTZ administration is on cycle days 3-7 (6).

Hormonal profile of LTZ cycles in infertility literature is a nowadays matter of challenge. It has been shown that LTZ can induce a marked decrease in plasma concentrations of estradiol (E_2_) and estrone, with approximately no effect on other steroidal hormones. No accumulation of androgens, androgen precursors, luteinizing hormone (LH), FSH, thyroid-stimulating hormone (TSH) or renin was reported in pharmacodynamics studies of LTZ (4, 8).

On the other hand, Garcia-Velasco et al. (9) in 2005 found significantly elevated follicular fluid levels of testosterone and androstenedione with LTZ therapy during ovarian stimulation for *in vitro* fertilization (IVF). Another study has reported significant higher LH, testosterone, androstenedione, and postovulatory progesterone (P) levels in LTZ treated patients compared to natural cycles (10). Also, in another research, some minor changes have been found in follicular phase hormonal profiles (P, LH, and E_2_) compared to natural cycles (11).

It seems that there are many unknown aspects of using aromatase inhibitors for ovulation induction. Thus, it is reasonable to do more studies. The aims of our study were to evaluate the cycle characteristics, including: follicular phase length, endometrial thickness, monofollicular response, and pregnancy rate, of LTZ in CC failure PCOS patients in order to compare fixed dose of LTZ between cycle days 3 and 5 and to evaluate the hormonal changes during these two protocols.

## Materials and Methods

Based on Rotterdam criteria, 70 PCOS patients were enrolled in this randomized clinical trial. A written informed consent was taken from all women participating in this study. The diagnosis of PCOS was made when two of the following three criteria existed: oligomenorrhea or amenorrhea, clinical hyperandrogenism, and polycystic ovaries on ultrasonography. The inclusion criteria were as follows: i. Previous diagnosis of PCOS according to Rotterdam criteria, ii. Age between 20-30 years, iii. No previous history of ovarian surgery and iv. lack of ovulation with CC in at least 3 previous cycles (lack of follicle ≥18 mm on ultrasound scan). The exclusion criteria were as follows: i. No other infertility factors, ii. Exposure to cytotoxic drugs and iii. Pelvic radiation therapy. The study was performed from March to November 2010 at the Mashhad IVF center, a university based infertility center.

The patient’s age, her partner’s age, duration of infertility, type of infertility (primary and secondary), history of previous intrauterine insemination (IUI) cycles, pattern of ovary (PCO and non-PCO), pattern of menstruation (regular, oligo-menorrhea and amenorrhea), body mass index (BMI) and basal LH/FSH ratio were recorded for each patient.

**Fig.1:**
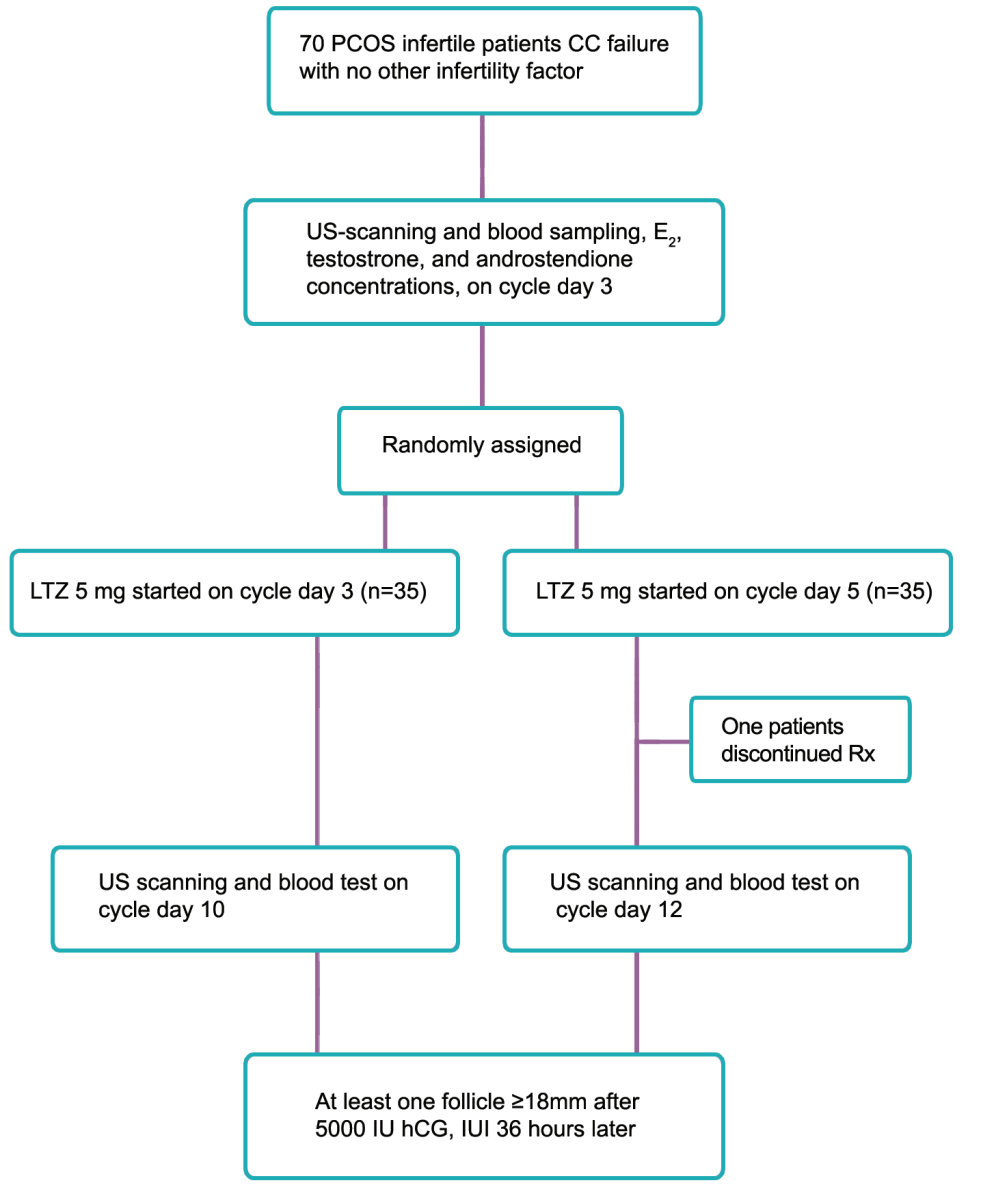
PCOS; Polycystic ovary syndrome, CC; Clomiphene citrate, E_2_; Estradiol , LTZ; Letrozole, hCG; Human chorionic gonadotropin and IUI; Intrauterine insemination.

The patients were randomly assigned into two groups: Group A (n=35) receiving 5 mg LTZ (Letrofem; Iran Hormone, Iran) on cycle days 3-7 (CD3), and group B (n=35) receiving the same amount on cycle days 5-9 (CD5) (Fig .1).

Both groups underwent a vaginal ultrasonographic (US) scan (probe 7.5 MHz; Ultrasonix, USA) and a blood analysis just before the first dose of LTZ and three days after the last dose of LTZ therapy. US scanning were continued if indicated.

By observing at least one follicle ≥18 mm, the patient was considered ovulatory and 5,000 -10,000 IU human chorionic gonadotropin (hCG) was injected followed by a single IUI 36-40 hours later. Pregnancy was documented by observing fetal pole 2 weeks after missed period.

The number of follicles .18 mm and the endometrial thickness were measured on US scanning. The length of follicular phase and the hormonal levels of LH, E_2_, testosterone, and androstendione were registered for all the patients before and three days after completion of LTZ.

We used t test, chi-square, Fisher’s exact test, and Mann-Whitney U test by SPSS (SPSS Inc., Chicago, IL, USA) version 12.0 for statistical analysis. A p value less than 0.05 was considered significant. A comparison was done between demographic characteristics in ovulatory (n=28) and anovulatory patients (n=41). Follicle numbers, endometrial thickness, follicular phase length and pregnancy rate were compared between CD3 and CD5 patients. The hormonal levels were compared between two groups before and after receiving LTZ. The hormonal levels were compared in ovulatory and anovulatory patients before and after treatment. This study was approved by Ethical Committee of Mashhad University of Medical Sciences.

## Results

The total number of recruited PCOS patient in this study was 70 (n=35/each group). There was no significant difference between two therapeutic groups considering patient’s characteristics, like: age, duration of infertility, pattern of ovary, BMI, and basal LH/ FSH ratio (Table 1).

The ovulation rate (presence of at least 1 follicle ≥18 mm during ovarian stimulation) in CD3 and CD5 groups were 48.6% (17/35) versus 32.4% (11/35), respectively, whereas the difference was not statistically significant (p=0.17).

The age, duration of infertility, pattern of ovary, BMI, and basal LH/FSH ratio were not statistically significant difference between ovulatory and anovulatory patients (Table 2).

By comparing the basal androgen level, we found that the patients with a successful ovulation as compared to anovulatory patients had a significant lower androstenedione (94.76±59.42 vs. 181.95±239.58 ng/dl, p=0.02) and testosterone (33.76±13.26 vs. 42.10±18.90 ng/dl, p=0.02) levels before treatment (Table 3).

The E_2_ concentration was similar between ovulatory and anovulatory patients before the treatment (56.66±29.02 vs. 65.54±26.93 pg/ ml, respectively, p=0.32), but it was significantly higher in ovulatory patients after treatment (118.35±72.89 vs. 56.18±46.13 pg/ml respectively, p=0.01, Table 3).

There was no significant difference between two therapeutic protocols regarding the cycle characteristics. Thus, follicular phase length (14.1±3.8 in CD3 and 14.7±1 days in CD5 patients), endometrial thickness (8.0±1.16 mm in CD3 and 7.8±1.3 mm in CD5 patients) and mono-follicular response (76% in CD3 and 82% in CD5 patients) were similar between groups (p>0.05, Table 4).

Between two groups, testosterone significantly increased during the treatment (36.55±34.55 ng/dl for CD3 and 24.71±28.20 ng/dl for CD5, p<0.01 for both). Although this increase was higher in CD3 patients, there is no statistically significant difference between two groups (p=0.15, Table 5).

Androstenedione was also increased during treatment course (75.72±151.92 ng/ml for CD3 and 119.50±116.27 ng/dl for CD5, p=0.01 for both). Although this increase was higher in CD5 patients, there is no statistically significant difference between two groups (p=0.21, Table 5).

The E_2_ pattern was different between the groups. Although there was a significant increase in the CD3 patients, (the change from baseline=31.24 ± 75.05 pg/ml, p=0.03), there was a decrease among the CD5 (the change from baseline=-8.96±42.64 pg/ml, p=0.24). So, E_2_ changes in two protocols showed statistically significant difference (p=0.01, Table 5).

The positive clinical pregnancy outcome was also higher in CD3 patients (12.1% in CD3 versus 9.4% in CD5 patients), but the difference was not statistically significant (p~1, Table 6).

**Table 1:** Basic and demographic characteristics in study groups


Variable		Treatment group		P value
		CD3	CD5	

**Age (Y)**		25.3± 4.4	25.6± 3.5	0.777
**BMI (kg/m^2^)**		27.0± 3.8	26.4± 4.81	0.674
**Types of infertility**	Primary	31 (91.2%)	34 (97.1%)	0.356
	Secondary	3 (8.8%)	1 (2.9%)	
**Pattern of ovary**	PCOS	24 (68.6%)	23 (65.7%)	1
	non-PCOS	11 (31.4%)	12 (34.3%)	
**Familial history of PCOS**	Yes	1 (2.9%)	3 (8.6%)	0.614
	No	34 (97.1%)	32 (91.4%)	
**LH /FSH (mIU/ml)**		1.35± 2.43	1.94± 1.91	0.082
**TSH (μu/ml)**		2.95± 3.03	3.29± 4.17	0.376^a^
**PRL (ng/ml)**		24.5± 41.38	29.2± 65.43	0.672^a^
**The number of previous treatment cycles**		1.09± 0.39	1.25± 0.52	0.096^a^


CD3; Cycle day 3, CD5; Cycle day 5, BMI; Body mass index, PCOS; Polycystic ovary syndrome, LH; Luteinizing hormone, FSH; Folliclestimulating hormone, TSH; Thyroid-stimulating hormone, PRL; Prolactin and a; Mann-Whitney U test results.

**Table 2:** Basic and demographic characteristics in ovulatory and non-ovulatory patients


Variable		Treatment group		P value
		Ovulatoty N=28	Anovulatory N=41	

**Age (Y)**		25.40±3.4	23.75±0.54	0.971	
**BMI (kg/m^2^)**		26.51±4.61	26.76±4.34	0.870	
**Types of infertility**	Primary	28 (100%)	37 (90.5%)	0.303	
	Secondary	0 (0%)	4 (9.5%)		
**PCO pattern in ovary by ultrasonography**	PCOS	20 (71.0%)	32 (78.0%)	0.600	
	non-PCOS	8 (29.0%)	9 (22.0%)		
**Familial history of PCOS**	Yes	1 (4.0%)	2 (5.0%)	0.303	
	No	27 (96.0%)	39 (95.0%)		
**LH (mIU/ml)**		10.17±7.37	10.30±6.49	0.942	
**FSH (mIU/ml)**		7.18±3.62	6.28±2.73	0.267	
**Menstrual pattern(n)**	Oligo-menorrhea	25 (90.5%)	28 (72.3%)	0.096	
	Amenorrhea	3 (9.5%)	13 (27.7%)


BMI; Body mass index, PCOS; Polycystic ovary syndrome, LH; Luteinizing hormone and FSH; Follicle-stimulating hormone.

**Table 3:** Comparison of follicular phase testosterone, androstendione, and estradiol dynamics in CD3 and CD5 patients, before and after treatment


Hormone	Blood sample	Ovulatory	Anovulatory	P value

**Testosterone (ng/dl)**	Before treatment	33.76±13.26	42.10±18.90	0.02
	After treatment	63.41±30.22	69.29±37.15	0.52
**Androstendione (ng/dl)**	Before treatment	94.76±59.42	181.95±239.58	0.02
	After treatment	176.35±90.92	289.159±207.78	0.00
**Estradiol (pg/ml)**	Before treatment	56.66±29.02	65.54±26.93	0.32
	After treatment	118.35±72.89	56.18±46.13	0.01


CD3; Cycle day 3 and CD5; Cycle day 5.

**Table 4:** Comparison of some cycle parameters in CD3 and CD5 patients


Cycle characteristics	CD3	CD5	P value

**Follicular phase length (day)**	14.1±3.8	14.7±1	0.093
**Endometrial thickness (mm)**	8.0±1.16	7.8±1.3	0.721
**Monofollicular response**	13 (76%)	9 (82%)	0.322
**(among ovulatory patients)**			


CD3; Cycle day 3 and CD5; Cycle day 5.

**Table 5:** Comparison of follicular phase testosterone, androstendione, and estradiol dynamics in CD3 and CD5 patients


Type of hormone	Group	Before treatment	After treatment	Difference	P value

**Testosterone (ng/dl)**	CD3	36.00±15.49	72.55±35.94	36.55± 34.55	0.147
	CD5	43.87±24.10	68.59±39.88	24.71± 28.20	
**Androstendione (ng/dl)**	CD3	153.24±176.29	228.96±116.63	75.72± 151.92	0.209
	CD5	169.78±244.52	289.28±232.78	119.50±116.27	
**Estradiol (pg/ml)**	CD3	64.00±29.36	95.24±76.94	31.24± 75.05	0.012
	CD5	64.03±25.55	55.06±31.96	-8.96±42.64	


CD3; Cycle day 3 and CD5; Cycle day 5. P values were calculated for the differences in hormonal values between CD3 and CD5.

**Table 6:** Comparison of cycle outcome in CD3 and CD5 groups


		CD3	CD5	P value

**Ovulation rate (at least one follicle more than 18 mm)**	Positive	17 (48.6%)	11 (32.4%)	0.174
**Clinical pregnancy**	Positive	4 (12.1%)	3 (9.4%)	1


CD3; Cycle day 3 and CD5; Cycle day 5.

## Discussion

In two different protocols of LTZ starting on cycle days 3 and 5, there were no significant differences in follicular phase length, endometrial thickness, monofollicular response, and pregnancy rate.

AIs were originally developed for the treatment of advanced breast cancer (12); however, it has been also introduced as reproductive medicine by Mitwally and Casper (7). They showed that LTZ was effective in inducing ovulation in women with PCOS. LTZ is rapidly and completely absorbed from gastrointestinal (GI) tract with absolute bioavailability of 99.9%. The terminal elimination half-life in plasma is about 2 days and the maximal suppression of estrogen concentration is achieved in 48-78 hours after single oral dose administration.

Since only a decade has passed since the introduction of LTZ in the field of ovarian stimulation, there is still debate about the optimal protocol to use. The dosage of LTZ, therefore, differs between studies. The majority of researchers have used 2.5-5.0 mg LTZ daily based on the dosage used for the treatment of patients with breast cancer (12). In one study, the effect of a single dose of 20 mg LTZ on cycle day 3 was compared to 2.5 mg on cycle days 3-7, which was not significantly different in pregnancy rate (13). In another study, 7.5 mg LTZ for 5 days was compared to 150 mg CC that was proved to be more efficacious in terms of ovulation and pregnancy rates (14).

The duration of stimulation in most studies was similar to CC, namely 5 days in the early follicular phase, although longer stimulation for 10 days has been also tested (15).

To induce ovulation, FSH is necessary in the early phase of the cycle to recruit and to select follicles. In ovulation cycles by gonadotropins, the earlier time for FSH administration is started during the cycles (prior to the selection phase) and more follicles are then recruited (11).

CC is usually administered on day 5 of menstruation. This is based on the theory that on cycle day 5, a physiologic decrease in FSH concentration provides the means for selection of the dominant follicle. Initiation of the drug on cycle day 2 induces earlier ovulation which is analogous to the physiologic events of the normal menstrual cycle. In one study, ovulation and conception rates and pregnancy outcome were similar when CC treatment started anywhere between cycle days 2 and 5 (16). In another study, CC was started on cycle days 3, 4, 5, or 7 in IVF cycles. The researchers concluded that protocol of cycle day 5 had more oocytes recovered, fertilized and transferred (17). In another study, CC commenced on day 1 of the menstrual cycle rather than day 5 resulted in more rapid follicular growth and higher pregnancy rate in IUI cycles (18). Treatment with CC was associated with higher rate of pregnancy if started early (days 1 through 5 than 5 through 9) in the menstrual cycle in the study by Dehbashi et al. (19).

Based on experiences on CC, the starting day for LTZ in most studies has been found on day 3 of spontaneous or induced menstruation. In one study, 5 mg LTZ administered on cycle days 1-10 showed higher pregnancy rate compared to same amount administered from cycle days 1-5 (1). In another study, they compared the effect of 2.5 mg LTZ administered on cycle days 2-6 to placebo and their findings showed 33.3% ovulation rate compared to 0.00% for the placebo (20).

In our study, we compared the cycle characteristics for two different starting days including cycle days 5 and 3 in CC resistant PCOS patients who developed no dominant follicle during their previous cycles with CC, examined by ultrasound scanning. Our findings did not show significant difference in ovulation rate between CD3 and CD5 groups. The overall ovulation rate was 40.6%. The monofollicular response showed no significant differences between two groups. In different studies, this rate has been reported between 33.3% (20) to 84.4% (21).

In our study, the endometrial thickness was not significantly different between CD3 and CD5 groups. The overall endometrial thickness in both groups was 7.9 mm compared to 11.2 mm in a study by Bedaiwy et al. (11) and 7.1 mm in a study by Al-Fozan et al. (14).

There was 4 (12.1%) clinical pregnancies in CD3 group and 3 (9.4%) in CD5 group. The total pregnancy rate was 10.8%. The pregnancy rate in cycles of LTZ treatment in literature is 5.6% (20) to 40.6% 9 (22).

In our research, we studied the hormonal dynamics as well. In both groups, testosterone and androstenedione concentrations were increased from baseline three days after termination of LTZ treatment. The change from baseline was 30.5 ng/dl for testosterone and 98.7 ng/dl for androstenedione. It could be realized that the patients with lower serum androgens level would get more benefit from LTZ treatment.

Serum E_2_ concentration was increased from baseline for 31.2 pg/ml in CD3 group and decreased for 8.96 pg/ml in CD5 group. This difference should be related to E_2_ secretion from the ovum.

Reviewing the pharmacokinetic studies of LTZ, there was no effect on plasma concentration of testosterone and androstenedione after single doses of 0.1-5 mg (8). On the other hand, Garcia-Velasco et al. (9) have shown a significant increase in intrafollicular androgen levels in IVF cycles treated by LTZ. Cortinez et al. (10) have also shown an increase in serum androgen level and a decrease in E_2_ levels on the last day of LTZ treatment compared to natural cycle.

In our study, the androgen levels were increased significantly 3 days after termination of LTZ, days 10 and 12 for CD3 and CD5 groups, respectively. The follicular phase length was shown in both groups for about 14 days, and high androgen level at the time of conception was also considered.

PCOS patients experience a higher incidence of miscarriage, preterm delivery and low birth weight infants (23) that has been attributed to hyperandrogenic state of this syndrome, indicated by many authors (24-26). The other effect of hyperandrogenism in PCOS pregnant patients at the early embryonic stage has been proposed as a developmental etiology for PCOS in female fetus (27).

Therefore, it seems logical that an increase in androgen level after receiving LTZ in hyperandrogenic PCOS patients at the time of conception should become a major concern.

Although we have not measured free androgen index (FAI) in our patients, but our finding showed that anovulatory patients in both groups had significantly higher basal androgen levels and this was in accordance with Imani et al. (28), in which they proved FAI as an important predictor of ovulation in CC protocol.

## Conclusion

LTZ is an alternative treatment in PCOS patients. Two different protocols of LTZ starting on cycle days 3 and 5 showed no significant differences in follicular phase length, endometrial thickness, monofollicular response, and pregnancy rate. Androgen levels were significantly increased after treatment. Lower basal androgen levels showed significantly better result. More studies are needed to evaluate different initiation day, length, and dose for LTZ administration.
